# Association between the p.V37I variant of *GJB2* and hearing loss: a pedigree and meta-analysis

**DOI:** 10.18632/oncotarget.17325

**Published:** 2017-04-21

**Authors:** Na Shen, Jing Peng, Xiong Wang, Yaowu Zhu, Weiyong Liu, Aiguo Liu, Yanjun Lu

**Affiliations:** ^1^ Department of Laboratory Medicine, Tongji Hospital, Tongji Medical College, Huazhong University of Science and Technology, Wuhan 430030, China; ^2^ Department of Otorhinolaryngology, Tongji Hospital, Tongji Medical College, Huazhong University of Science and Technology, Wuhan 430030, China

**Keywords:** hearing loss (HL), *GJB2*, p.V37I, pedigree analysis, meta-analysis

## Abstract

Pathogenic variants in the gap junction protein beta-2 (*GJB2*) gene are the most common cause of hearing loss. Of these, the p.V37I variant of *GJB2* has a high allele frequency (up to 10%) in East Asians. Characterization of the phenotypic spectrum associated with p.V37I, as well as the role of this variant in the onset of hearing loss could have a remarkable effect on future diagnostic strategies. Here, we performed a pedigree analysis of unrelated families exhibiting various hearing phenotypes, and then conducted a meta-analysis to comprehensively assess the association between the p.V37I and the risk of hearing loss. Pedigree analyses showed that both homozygous p.V37I variants, as well as compound heterozygous p.V37I with other *GJB2* pathogenic variants, contributed to various phenotypes of hearing loss. Meanwhile, meta-analysis demonstrated that, compared with those in the wild type group, both p.V37I homozygotes and compound heterozygous p.V37I variants were at significantly higher risk of developing hearing loss (odds ratios = 7.14 and 3.63; 95% confidence intervals = 3.01-16.95 and 1.38–9.54, respectively). Conversely, heterozygous p.V37I variants alone did not increase the risk of hearing loss. Given the high allele carriage rate of p.V37I (up to 10%) within the general population, our work not only provides information that might influence future genetic screening policies, but also offers insight into clinical risk evaluation and genetic counseling regarding hearing loss.

## INTRODUCTION

Hearing loss (HL) is the most sensory defect that affects 1-3 in every 1,000 newborns worldwide, and half of these cases are attributed to genetic factors [1]. Notably, while a large number of HL-related genes have been identified, the *gap junction protein beta 2* (*GJB2*) gene accounts for nearly 20% of all cases of HL, as well as 50% of autosomal recessive non-syndromic HL, in many populations [2, 3]. *GJB2* encodes the connexin 26 protein, which comprises a critical component of cochlear gap junctions, and is important to cell communication. To date, greater than 300 variants within *GJB2* have been found to be associated with HL (http://www.hgmd.cf.ac.uk/ac/), including c.35delG, c.235delC, and c.176_191del16.

In particular, the p.V37I (c.109G>A) variant of *GJB2* has a high allele frequency (up to 10%) among East Asian populations [4–6]. This variant, harboring a missense substitution from valine to isoleucine at codon 37, was first identified by Kelley et al. in 1998 [7]. Early studies regarded the p.V37I as a benign polymorphism, as it was observed in unaffected heterozygous controls [7–11]. However, the identification of increasing numbers of HL patients that are homozygous for p.V37I, or compound heterozygous for p.V37I and other *GJB2* pathogenic variants, indicates that p.V37I likely increases the risk of HL, particularly for mild-to-moderate cases [6, 12–15]. Interestingly, a recent meta-analysis reported an insignificant association between the carriage rates of p.V37I and HL, which aroused wide concern regarding the pathogenicity of this variant [16].

Given the high allelic frequency of the p.V37I variant (up to 10%), it is estimated that greater than five million East Asians suffer from HL due to homozygous or compound heterozygous carriage of this allele [6]. It is therefore imperative to evaluate the risks associated with p.V37I for clinical genetic counseling and public health assessment purposes. In this study, we performed a pedigree analysis of families with probands exhibiting various HL phenotypes, and then conducted a meta-analysis of sporadic HL to comprehensively evaluate the role of p.V37I in the risk of HL.

## RESULTS

### Pedigree analyses

**Figure 1:**
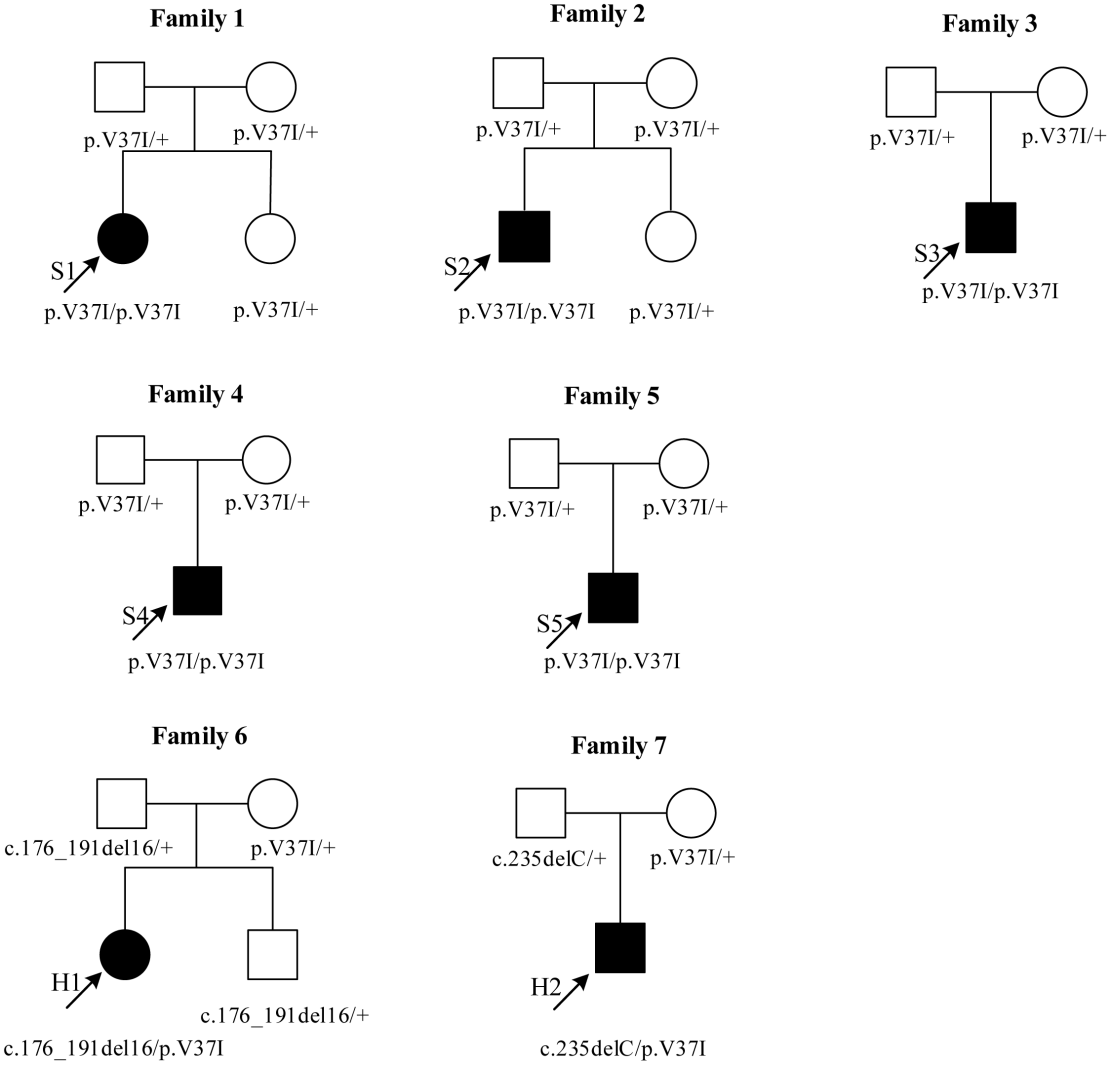
Pedigree analyses for seven unrelated families carrying the p.V37I variant Five probands (S1-S5) carried two p.V37I alleles as homozygotes. Two probands (H1, H2) carried the p.V37I allele in a trans configuration with another well-known pathogenic mutant allele of *GJB2*, as compound heterozygotes. The hearing level of S1 was recorded by auditory brainstem responses (ABR) due to her very young age (7 months). The hearing level of H2 was recorded by the auditory steady-state responses (ASSR) because of his poor cooperation in pure-tone audiometry (PTA) test. Sanger sequencing results of pedigree analyses were shown in Supplementary Figure 4.

Seven families carrying the p.V37I variant were included for pedigree analyses (Table 1 and Figure 1). There were five male and two female probands. Six probands were children (7 months-9 years) and one proband was an adult (33 years). These probands exhibited both congenital and delayed-onset non-syndromic HL, and one member had sudden deafness. Bilateral and unilateral non-syndromic HL were also observed, with HL degrees of mild to moderate. Families 1–5 had probands that were homozygous for p.V37I (S1–S5), while the probands of Families 6 and 7 had compound heterozygous p.V37I variants (H1–H2). In this study, compound heterozygous p.V37I variants were defined as the p.V37I allele in a trans configuration with another pathogenic mutant allele of *GJB2* gene. Pedigree analyses revealed that p.V37I was transmitted from heterozygous parents to their children, who suffered HL if he/she inherited two affected alleles (p.V37I homozygotes or compound heterozygous p.V37I variants). Meanwhile, siblings that inherited one affected allele retained normal hearing. These analyses strongly suggest that p.V37I increases the risk of HL through an autosomal recessive inheritance pattern.

**Table 1:** Clinical characteristics of probands carrying the p.V37I variant

ID	Sex	Age	p.V37I status	Onset	Site	Degree
S1	Female	7 months	Homozygote	Congenital	Bilateral	Moderate
S2	Male	8 years	Homozygote	Congenital	Bilateral	Mild
S3	Male	9 years	Homozygote	Congenital	Unilateral	Mild
S4	Male	33 years	Homozygote	Delayed-onset	Bilateral	Moderate
S5^a^	Male	8 years	Homozygote	Sudden deafness	Bilateral	Mild
H1	Female	7 years	p.V37I/c.176_191del16	Delayed-onset	Bilateral	Mild
H2	Male	3 years	p.V37I/c.235delC	Congenital	Bilateral	Moderate

^a^ The severity of deafness of proband S5 was 30 dB at the left ear, and 31.25 dB at the right ear, so his degree of HL was considered as mild.

### Meta-analysis on sporadic HL

To further determine the detrimental effects of p.V37I, we performed a meta-analysis. A flow chart of the literature search is shown in Supplementary Figure 1. A total of 1,085 potentially relevant records were initially identified in our search. Of these, 391 duplicates and 644 irrelevant records were removed upon reviewing titles and abstracts, yielding 50 full-text articles for further evaluation. Seventeen studies were subsequently excluded because they provided no genetic data regarding the p.V37I variant (n = 7), they lacked sufficient information to estimate odds ratio (OR) and 95% confidence interval (CI) (n = 7), they lacked a parallel control group (n = 2), or their data overlapped with that of another study (n = 1). Of the 33 remaining studies eligible for meta-analysis [5, 7–15, 17–39], 10 also provided genotypes of compound heterozygous p.V37I variants [8, 12, 13, 19, 21, 28, 33–35, 37].

**Figure 2:**
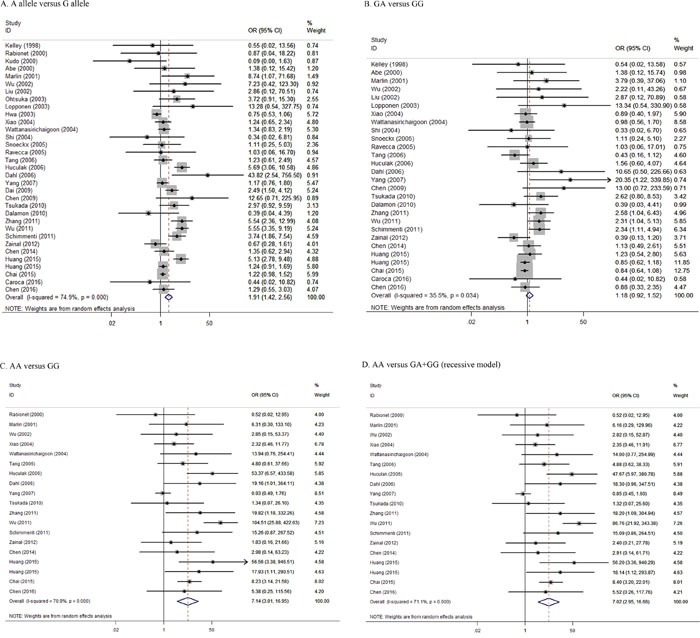
Forest plots of the effects of p.V37I on HL risk under the (A) allelic, (B and C) codominant, and (D) recessive models Allelic model referred to A allele versus G allele. Codominant model referred to GA genotype versus GG genotype **(B)**, or AA genotype versus GG genotype **(C)**. Recessive model referred to AA genotype versus GA+GG genotype.

As shown in Table 2, a total of 14,398 HL cases and 8,699 controls were included to evaluate the association between p.V37I and HL risk. Twenty of the studies were conducted in Asia (China, Japan, Malaysia and Indonesia) [5, 8, 9, 12–14, 18, 20, 22–24, 26, 30, 31, 34–37, 39], while six were conducted in North America (United States and Canada) [7, 11, 15, 19, 28, 33], five in Europe (Portugal, Italy, Finland, France and Spain) [10, 17, 21, 25, 38], one in Latin America (Argentina) [32], and one in Oceania (Australia) [27]. Overall, there was a significant association between the p.V37I variant and increased risk of developing HL (Figure 2). Specifically, the A allele of p.V37I was associated with a 2-fold higher risk of developing HL than the G allele (Figure 2A; OR = 1.91; 95% CI = 1.42–2.56; *P_heterogeneity_* < 0.001; *I^2^* = 74.9%). Moreover, compared with individuals with wild type, p.V37I homozygotes (Figure 2C; OR = 7.14; 95% CI = 3.01-16.95; *P_heterogeneity_* < 0.001; *I^2^* = 70.9%), but not p.V37I heterozygotes (Figure 2B; OR = 1.18; 95% CI = 0.92–1.52; *P_heterogeneity_* = 0.034; *I^2^* = 35.5%), had a significantly higher risk (7.14-fold greater) of developing HL. Similar results were obtained using the recessive model (Figure 2D; OR = 7.02; 95% CI = 2.95–16.66; *P_heterogeneity_* < 0.001; *I^2^* = 71.1%). Sensitivity analyses demonstrated that our results were quite stable (Supplementary Figure 2A–2D) and no obvious publication biases were found (all *P* > 0.10).

**Table 2:** Characteristics of studies included for meta-analysis

First author	Publication year	Country	Geological area	Population	Cases	Controls	*P_HWE_*
Chen [39]	2016	China	Asia	Children and adults	50	53	0.399
Caroça [38]	2016	Portugal	Europe	Children and adults	134	177	0.970
Huang [12]	2015	Shanghai, China	Asia	Infants	300	484	0.747
Huang [13]	2015	China	Asia	Infants and adults	3,864	600	0.318
Chai [14]	2015	China	Asia	Infants and adults	945	1,550	0.557
Chen [37]	2014	China	Asia	Infants and child	107	61	0.486
Zainal [36]	2012	Malaysia	Asia	Children	32	37	0.479
Zhang [35]	2011	China	Asia	Children and adults	236	107	0.765
Wu [34]	2011	China	Asia	Infants	38	979	0.139
Schimmenti [33]	2011	United States	North America	Infants	1,177	1,177	0.884
Tsukada [5]	2010	Japan	Asia	Infants and children	1,343	252	0.924
Dalamon [32]	2010	Argentina	Latin America	NR	252	50	0.943
Dai [31]	2009	China	Asia	Children and adults	1,372	301	NA
Chen [30]	2009	China	Asia	NR	115	109	NA
Yang [29]	2007	China	Asia	NR	260	120	<0.001
Tang [28]	2006	United States	North America	NR	610	294	0.004
Huculak [15]	2006	Canada	North America	NR	40	100	0.751
Dahl [27]	2006	Australia	Oceania	Children	48	90	NA
Snoeckx [26]	2005	Indonesia	Asia	Patients: <20 years old; Control: adults	120	100	0.879
Ravecca [25]	2005	Italy	Europe	Children and adults	39	40	0.936
Xiao [24]	2004	China	Asia	NR	131	100	0.100
Wattanasirichaigoon [11]	2004	United States	North America	Children and adults	166	205	0.181
Shi [23]	2004	China	Asia	Patients: infants; Control: NR	20	50	0.827
Ohtsuka [22]	2003	Japan	Asia	NR	1,227	147	NA
Lopponen [21]	2003	Finland	Europe	Patients: children; Control: NR	71	313	NA
Hwa [20]	2003	China	Asia	NR	324	432	NA
Wu [19]	2002	United States	North America	Patients: children; Control: NR	324	100	NA
Liu [18]	2002	China	Asia	Patients: children and adults; Control: NR	210	200	NA
Marlin [17]	2001	France	Europe	Patients: children; Control: NR	96	116	0.963
Rabionet [10]	2000	Italy and Spain	Europe	Patients: children; Control: NR	576	100	NA
Kudo [9]	2000	Japan	Asia	Patients: children and adults; Control: NR	78	63	NA
Abe [8]	2000	Japan	Asia	NR	35	96	0.918
Kelley [7]	1998	United States	North America	NR	58	96	0.959

NR: not reported; NA: not available; *P_HWE_*: *P* value for test of Hardy-Weinberg equilibrium.

**Figure 3:**
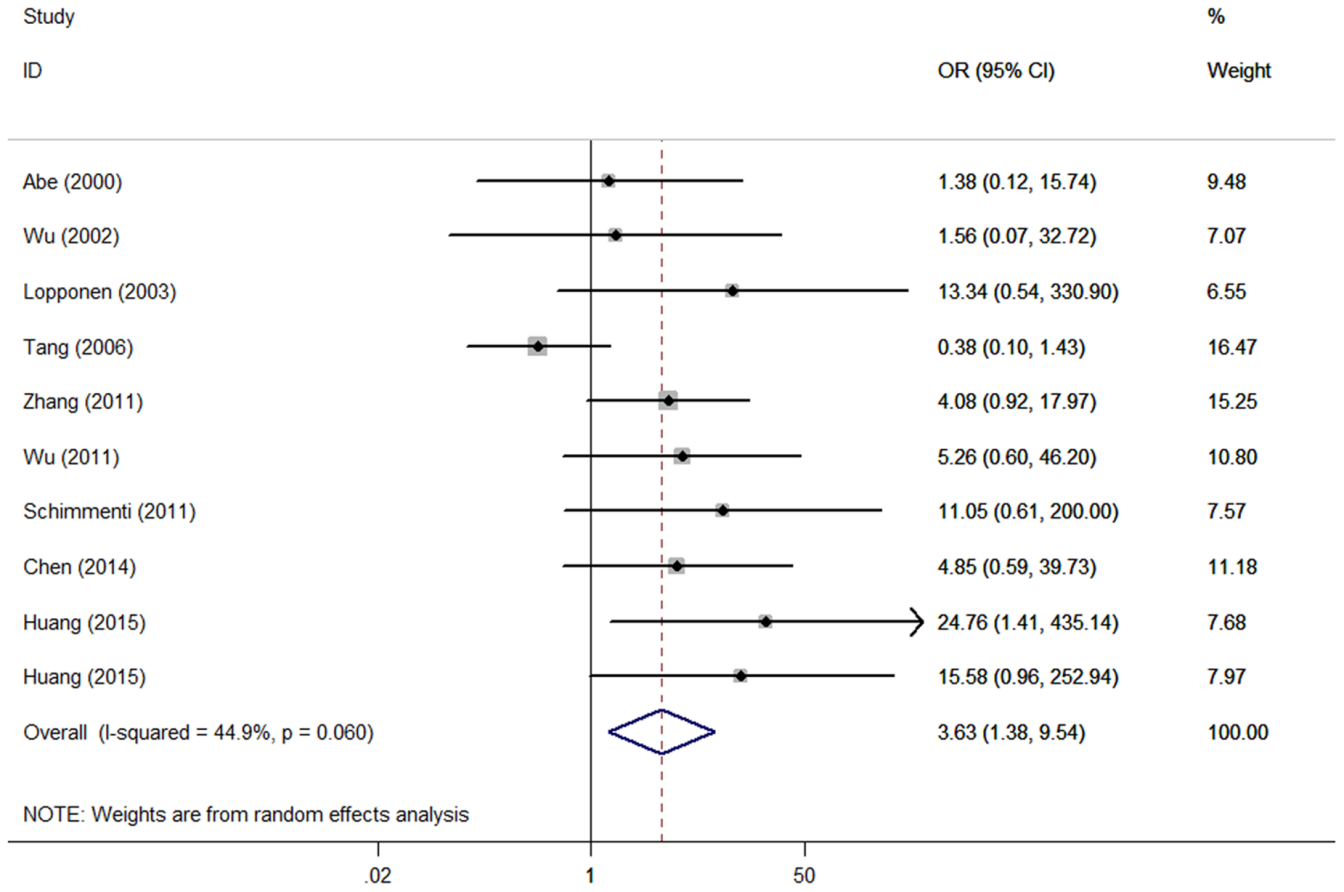
Forest plots of the effects of compound heterozygous p.V37I variants on HL risk Compound heterozygous p.V37I variants referred to the heterozygous p.V37I allele in a trans configuration with another well-known pathogenic mutant allele of *GJB2* gene.

Furthermore, we evaluated the association between compound heterozygous p.V37I variants on HL risk. Ten eligible studies comprising of 6,762 cases and 4,211 controls were included (Table 3). Notably, our results suggest that people harboring compound heterozygous p.V37I variants have a 3.63-fold higher risk of HL than those without these variants (Figure 3; OR = 3.63; 95% CI = 1.38–9.54; *P_heterogeneity_* = 0.060; *I^2^* = 44.9%). These results were strongly supported by sensitivity analyses (Supplementary Figure 3), and no publication bias was found (*P* = 0.152).

**Table 3:** Characteristics of studies for the association between compound heterozygous p.V37I variants and HL risk

First author	Publication year	Country	Population	Cases	Controls	Types
Huang [12]	2015	China	Infants	300	484	p.V37I/c.235delC, p.V37I/c.299_300delAT, p.V37I/c.79G>A, p.V37I/c.(79G>A+341A>G)
Huang [13]	2015	China	Infants and adults	3,864	600	p.V37I/c.235delC, p.V37I/c.299delAT, p.V37I/p.R143W, p.V37I/c.176del16, p.V37I/c.512insAACG, p.V37I/p.T86R, p.V37I/p.W77*
Chen [37]	2014	China	Infants and child	107	61	p.V37I/c.235delC, p.V37I/c.608T>C, p.V37I/c.(79G>A; 341A>G)
Zhang [35]	2011	China	Children and adults	236	107	p.V37I/c.235delC, p.V37I/c.427C>T, p.V27I/p.V37I/p.E114G, p.V27I/p.V37I, p.V37I/p.I203T, p.V27I/p.V37I/p.V84M
Wu [34]	2011	China	Infants	38	979	p.V37I/c.235delC
Schimmenti [33]	2011	United States	Infants	1,177	1,177	p.V37I/p.L90P, p.V37I/p.(V27I, E114G)
Tang [28]	2006	United States	NR	610	294	p.V37I/c.35delG, p.V37I/p.L90P, p.V37I/p.R216fsX232, p.V37I/p.V27I, p.V37I/(p.V27I+p.E114G), p.V37I/p.I203T
Lopponen [21]	2003	Finland	Patients: children; Control: NR	71	313	p.V37I/p.M34T
Wu [19]	2002	United States	Patients: children; Control: NR	324	100	p.V37I/p.M34T, p.V37I/c.167delT
Abe [8]	2000	Japan	NR	35	96	p.V37I/p.R143W, p.V37I/c.235delC

NR: not reported.

## DISCUSSION

In this study, we demonstrated that bi-allelic or compound heterozygous p.V37I variants are associated with increased risk of various HL phenotypes, and quantified the risk associated with these variants and development of HL.

Our pedigree analyses indicated that p.V37I can cause HL as either a homozygous variant or as compound heterozygous with other pathogenic variants in *GJB2*. Functional studies performed in cells and mouse models support this conclusion [40–42]. In consistent with previous reports [6, 12–14], we observed that the HL phenotypes associated with p.V37I varied by onset type, disease site, and degree of HL, implying that other causes, especially environmental factors, influence p.V37I-mediated onset of HL. Notably, we also found that homozygous p.V37I might give rise to sudden deafness in children. Indeed, similar results were reported in a recent study [39]. As such, p.V37I might be considered as a potential cause of sudden onset of deafness in future cases.

To quantify the pathogenic association between p.V37I and HL risk, we performed a meta-analysis. In keeping with previous cohort studies and functional experiments, our results supported the conclusion that the p.V37I variant significantly increases an individual's risk of developing HL. Compared with wild type individuals, the p.V37I homozygote group, but not the heterozygote group, showed a significantly greater likelihood of developing HL. This could explain why p.V37I heterozygotes are prevalent among the general healthy population, while bi-allelic p.V37I variants are found predominantly in patients with HL. Notably, our results also indicate that the compound heterozygous p.V37I variants are associated with a nearly four-fold greater risk of developing HL, compared to wild type individuals. In view of the high prevalence of p.V37I and the large number of other *GJB2* pathogenic variants, our findings indicate that previous reports have likely underestimated HL risk in human populations. Compared with the previous meta-analysis [16], our work included more eligible studies and quantified the risky effects of p.V37I on HL in more detail.

The major strengths of our work were the variable phenotypic spectrum associated with p.V37I and that a large population (23,097 participants) was used to evaluate the association between this variant and HL risk. However, our results should be interpreted with caution. First, only commonly known HL-related variants (variants within the *GJB2*, *SLC26A4*, *12S rRNA*, and *GJB3* coding regions) were screened in our pedigree analysis. Second, the number of studies regarding compound heterozygous p.V37I variants evaluated herein was insufficient to further explore the concrete effects of particular compound variant types on HL risk. Third, although both our study and previous reports found that p.V37I is associated with various HL phenotypes, the specific mechanism by which this variant promotes HL, such as interactions between p.V37I and other genetic or environmental factors, remains unclear. Further studies are therefore needed to address these issues.

In summary, our work strongly suggests a pathogenic role for p.V37I in various HL phenotypes and provides a quantitative assessment of the risk associated with carriage of this variant and development of HL. Considering the high carriage rate of p.V37I within the general population, these findings provide compelling information that should influence future genetic screening policy and that offer insights into clinical risk evaluation and genetic counseling.

## MATERIALS AND METHODS

### Study participants and ethical statement

For this study, we recruited seven unrelated probands with non-syndromic, sensorineural hearing loss, and their family members. Each participant provided written informed consent. For underage participants, written informed consent was obtained from their parents. Our study was approved by the Medical Ethics Committee of Tongji Hospital, Tongji Medical College, Huazhong University of Science and Technology. All procedures were performed according to the ethical guidelines for human subjects research.

### Clinical and audiometric evaluation

All participants were subjected to physical and neurological examinations to exclude syndromic deafness. Each participant's level of hearing was assessed via a comprehensive auditory evaluation, such as by otoscope examination, tympanometry, or pure-tone audiometry (PTA). For very young participants, auditory brainstem responses (ABR) and/or auditory steady-state responses (ASSR) were recorded. Degree of hearing loss was estimated as the average hearing levels at 0.5, 1.0, 2.0, and 4.0 kHz for the better ear. Severity of hearing loss was categorized as normal (<25 dB), mild (26-40 dB), moderate (41–70 dB), severe (71–95 dB), or profound ( >95 dB). In addition, we defined sudden deafness as over 30 dB of sensorineural hearing loss involving at least three frequencies occurring less than 72 hours [43].

### DNA extraction and mutation analysis

Genomic DNA was extracted from anticoagulant peripheral blood by the QIAamp DNA blood mini kit (Qiagen, Germany). For mutation screening, the entire coding region and flanking sequences of four commonly mutated genes, *GJB2*, *SLC26A4*, *12S rRNA*, and *GJB3*, were polymerase chain reaction (PCR) amplified and then subjected to bidirectional sequencing with an ABI 3500 DNA sequencer (Applied Biosystems). The primers and PCR conditions used for these analyses are described in detail in Supplementary Table 1. Sanger sequencing results of pedigree analyses were shown in Supplementary Figure 4.

### Meta-analysis on sporadic HL

We further performed a meta-analysis to determine the detrimental effects of p.V37I on sporadic HL, according to the guidelines of Preferred Reporting Items for Systematic Reviews and Meta-Analyses (PRISMA) [44]. A structured literature search was conducted of articles published through November 2016 using the PubMed, Web of Science, and EMBASE databases and the following search items: “*GJB2* OR connexin 26”, “polymorphism OR variant OR mutation”, “hearing loss OR deafness”, and “case-control OR cohort OR population”. Only English language articles were included. The inclusion criteria were: (i) studies that investigated the association between p.V37I and HL under a case-control or cohort design; (ii) studies that reported genotypes or allelic data for p.V37I to estimate OR and 95%CI. In cases of overlapping populations, the study with the largest sample size was included. We used OR as the effect measure and combined data using a random-effects model. Hardy-Weinberg equilibrium (HWE) in the controls was checked by χ^2^ test. Meanwhile, Cochran χ^2^ test and *I^2^* values were used to evaluate the heterogeneity between studies. Sensitivity analyses and publication bias assessment were also conducted. All statistical analyses were performed using Stata 12.1 software (StataCorp, College Station, TX, USA), and *P* ≤ 0.05 was considered significant for all tests.

## SUPPLEMENTARY MATERIALS FIGURES AND TABLE




