# Supplementary material for: Association between the p.V37I variant of GJB2 and hearing loss: a pedigree and meta-analysis
Source: Oncotarget. 2017 Apr 21;8(28):46681–90. doi: 10.18632/oncotarget.17325 (PMC5542302; doi:10.18632/oncotarget.17325)
Supplement: Supplementary file 1 [file oncotarget-08-46681-s001.pdf]

Association between the p.V37I variant of *GJB2* and hearing loss: a pedigree and meta-analysis

SUPPLEMENTARY MATERIALS

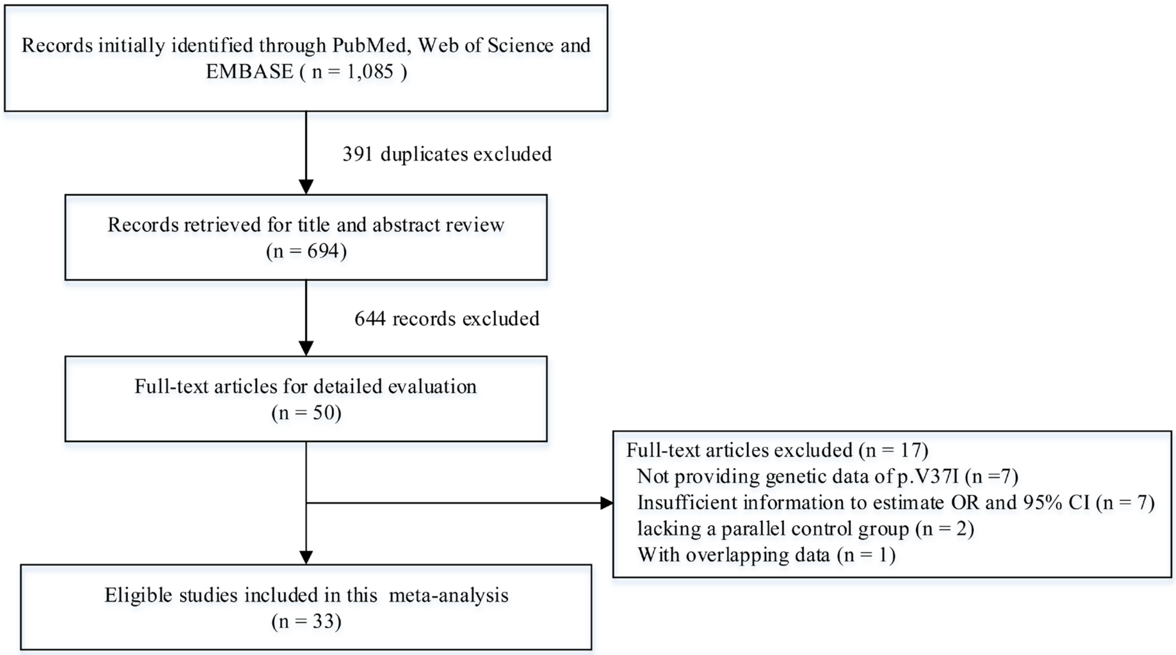

Supplementary Figure 1: A flow chart of literature search.

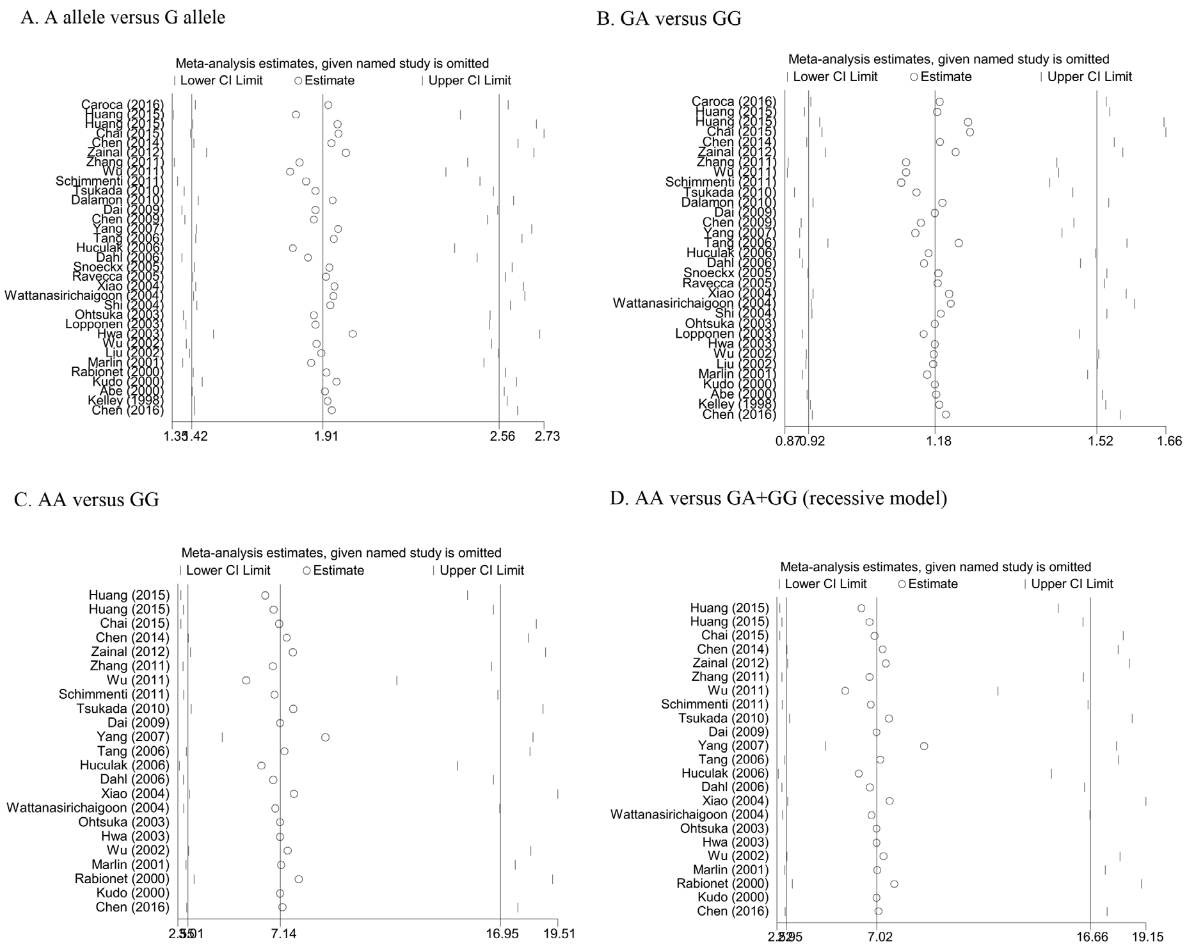

Supplementary Figure 2: Sensitivity analyses of the effects of p.V37I on HL risk under the (A) allelic, (B and C) codominant, and (D) recessive models.

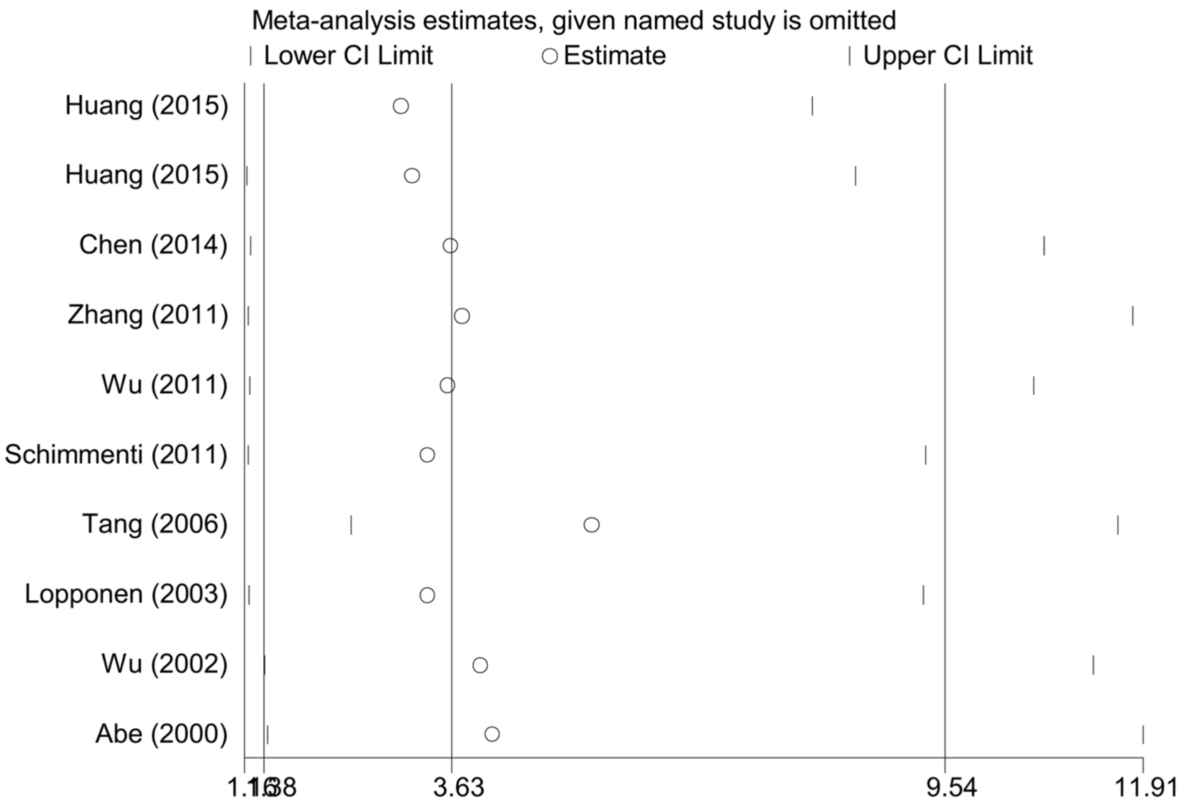

Supplementary Figure 3: Sensitivity analysis of the effects of compound heterozygous p.V37I variants on HL risk.

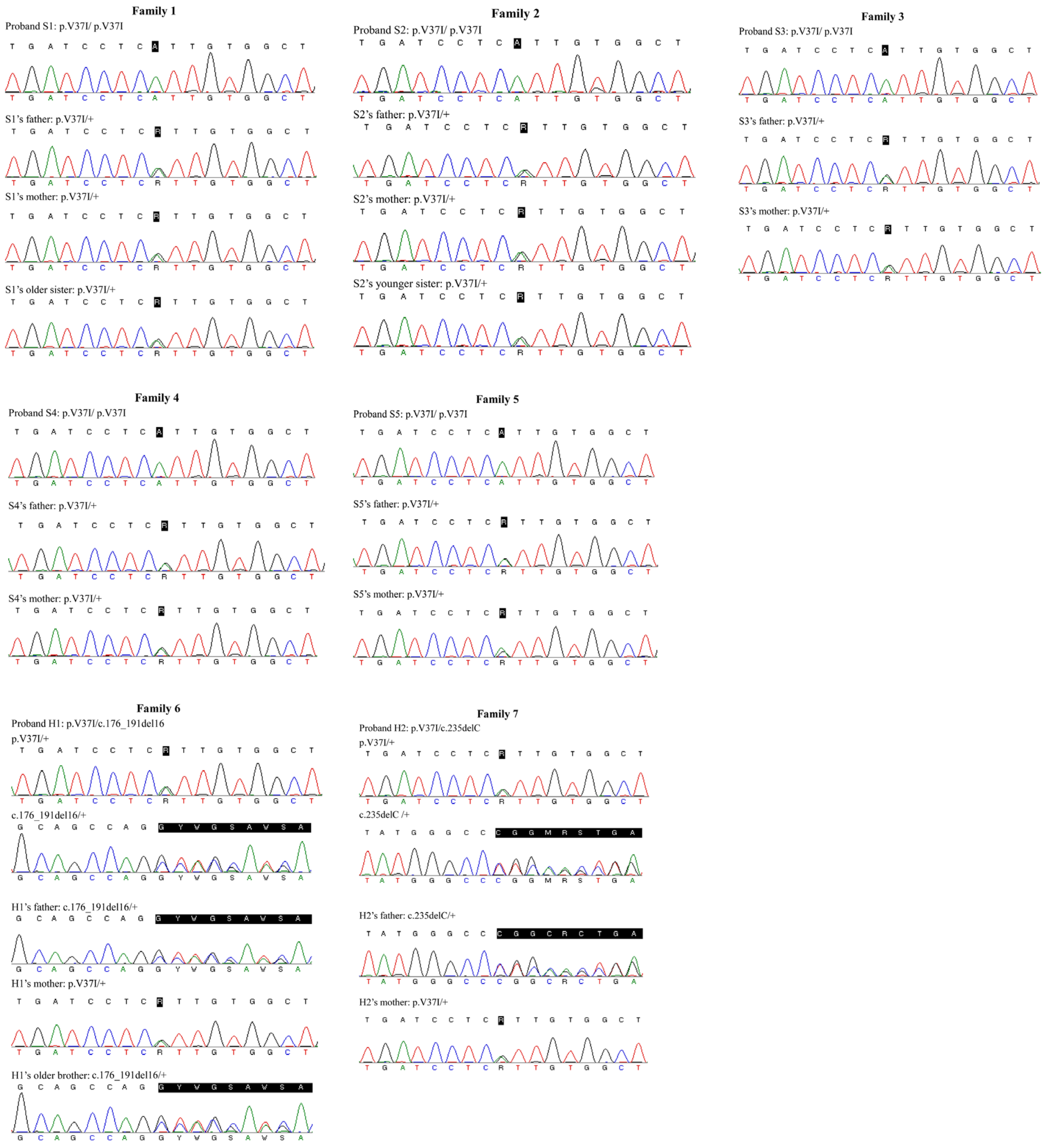

Supplementary Figure 4: Sanger sequencing results of pedigree analyses.

Supplementary Table 1. Primers and PCR conditions used for mutation analyses

See Supplementary File 1
