# Supplementary material for: Association between the p.V37I variant of GJB2 and hearing loss: a pedigree and meta-analysis
Source: Oncotarget. 2017 Apr 21;8(28):46681–90. doi: 10.18632/oncotarget.17325 (PMC5542302; doi:10.18632/oncotarget.17325)
Supplement: Supplementary file 2 [file oncotarget-08-46681-s002.doc]

Supplementary Table 1. Primers and PCR conditions used for mutation analyses

| Gene | PCR primer^b^ | Sequence (5'-3') | Product size (bp) | Sequencing primer |
| --- | --- | --- | --- | --- |
| *GJB2* (NM_004004.5) | G2-F | CGTTCAAGAGGGTTTGGGAACT | 1001 | G2-P1-F: ATTCCTGTGTTGTGTGCATTCG; G2-P1-R: GATGCGGACCTTCTGGGTTT; G2-P2-F: CCATGCACGTGGCCTACC; G2-P2-R: GCACGGGTTGCCTCATCC |
|  | G2-R | TGGGAAATGCTAGCGACTGA |  |  |
| *GJB3* (NM_024009.2) | G3-F | GTTCTGAACTCAGAGGGTCGTT | 1145 | G3-P1-F: TGCCTGGTACATAGTAAATGCTCA; G3-P1-R: GAAGCCATGCCAGAGAGTGT; G3-P2-F: CCTGTTCAGCCTCATCTTCA; G3-P2-R: GCCCTGCATGTCCCATT |
|  | G3-R | ACCTATACCCGGCTAGACAGC |  |  |
| *12s rRNA* (NC_012920.1) | 12S-F | CTCCATGCATTTGGTATTTTCGTC | 1916 | 12S-P1-F: CCCCATAAACAAATAGGTTTGGT; 12S-P1-R: TGTGGCTCGTAGTGTTCTGGC; 12S-P2-F: TTAGATACCCCACTATGCTTAGCC; 12S-P2-R: AGTGTAAGTTGGGTGCTTTGTGTTA |
|  | 12S-R | ACATAGACGGGTGTGCTCTTTTA |  |  |
| *SLC26A4* (NM_000441.1)^a^ | SLC-E2-F | GACCAGACTCGCGGTGCA | 525 | SLC-E2-F |
|  | SLC-E2-R | TTTCCCAGGTAAGTTCATTTCG |  | SLC-E2-R |
|  | SLC-E3-F | CACTTCAGGGTTATTATTTTCCAGG | 484 | SLC-E3-F |
|  | SLC-E3-R | CTAGTGGGGCAGCAGTTTCC |  | SLC-E3-R |
|  | SLC-E4-F | GCATCATCATAAAGGCAAAGTCA | 439 | SLC-E4-F |
|  | SLC-E4-R | GAATAGAAAAAGCAGGCAAAACACT |  | SLC-E4-R |
|  | SLC-E5-F | CAGCTTCTTTCGTGAACAAACAATA | 510 | SLC-E5-F |
|  | SLC-E5-R | AGTGAGCCTTAATAAGTGGGGTCTT |  | SLC-E5-R |
|  | SLC-E6-F | AGCTTGATGTAATATTTCCAGAGAG | 288 | SLC-E6-F |
|  | SLC-E6-R | GGAATGAACAGTGACCCATC |  | SLC-E6-R |
|  | SLC-E7E8-F | GGTTTTTCATGTGGGAAGATTCA | 593 | SLC-E7E8-F |
|  | SLC-E7E8-R | TATGGTTGTTTCTTCCAGATCACAC |  | SLC-E7E8-R |
|  | SLC-E9-F | GGACAAAGAAATCAGCCAGTAAGA | 504 | SLC-E9-F |
|  | SLC-E9-R | GCAAACACAAGAGGAAAACCAAC |  | SLC-E9-R |
|  | SLC-E10-F | TGGTATGGCGTCCAAACTCC | 464 | SLC-E10-F |
|  | SLC-E10-R | TCAGGTGAGGGAGTGGAACA |  | SLC-E10-R |
|  | SLC-E11E12-F | GGGAAGTATGAAGTGTGTCTGTGAA | 539 | SLC-E11E12-F |
|  | SLC-E11E12-R | GGAGTTCCCAAAGCACAAGTG |  | SLC-E11E12-R |
|  | SLC-E13-F | ATGTAATTTGTTTGTGGATCATTGAT | 466 | SLC-E13-F |
|  | SLC2-E13-R | ATCTTAGGGATGCTTCTTAGCTTTT |  | SLC2-E13-R |
|  | SLC-E14-F | AGAGAGGCACAGTTCTCCCC | 460 | SLC-E14-F |
|  | SLC-E14-R | TTCACATAAGCCATGGGTGTCA |  | SLC-E14-R |
|  | SLC-E15-F | AAAAGTTGAGTGCTGCTACCCA | 495 | SLC-E15-F |
|  | SLC-E15-R | TTTGAAACTTACTCAAATTCTGCCT |  | SLC-E15-R |
|  | SLC-E16-F | TCTGGGTAGGAGTAGGGTAGCC | 505 | SLC-E16-F |
|  | SLC-E16-R | AAAAAAATCACATCCTGTGTTTCC |  | SLC-E16-R |
|  | SLC-E17-F | AAATTCATCTCCTTGATGTCTTGC | 542 | SLC-E17-F |
|  | SLC-E17-R | CCAGTATCTCCCCCAGGTAGTCT |  | SLC-E17-R |
|  | SLC-E18-F | GAACCCGATAAGCATAGCCAC | 473 | SLC-E18-F |
|  | SLC-E18-R | TGACCACAGTCCCAGATAGG |  | SLC-E18-R |
|  | SLC-E19-F | GGCAATAGAATGAGACTCTGT | 312 | SLC-E19-F |
|  | SLC-E19-R | CTAGACTTGTGTAATGTTTGCC |  | SLC-E19-R |
|  | SLC-E20-F | TATACCAGTTCACCTTTCAATGTGC | 431 | SLC-E20-F |
|  | SLC-E20-R | TTTTTCCCTGCTATGAAGCCA |  | SLC-E20-R |
|  | SLC-E21-F | ACACCTAAGATGAGTAGCAGTAAG | 561 | SLC-E21-F |
|  | SLC-E21-R | GTGATCAGTGCAGCTCATGG |  | SLC-E21-R |

^a^ Primers are modified based on Prasad et al. (2004).

^b^ *GJB2*, *GJB3* and *12s rRNA* were amplified using TaKaRa LA Taq® (No. RR02MA) and the following PCR conditions: 30 cycles of 98℃ for 10 seconds, 60℃ for 30 seconds and 72℃ for 1 minute, ending with an extension cycle of 72℃ for 5 minutes. *SLC26A4* was amplified using TaKaRa Taq™ Hot Start Version (No. R007) and the following PCR conditions: 30 cycles of 98℃ for 10 seconds, 60℃ for 30 seconds and 72℃ for 45 seconds.
